# Longitudinal Links Between Perceived Family Support, Self-Efficacy, and Growth Mindset of Intelligence Among Chinese Children

**DOI:** 10.3390/bs15091182

**Published:** 2025-08-30

**Authors:** Wei Peng, Feng Zhang

**Affiliations:** 1 School of Public Affairs, Zhejiang Shuren University, Hangzhou 310015, China; 601476@zjsru.edu.cn; 2 Institute of Modern Services, Zhejiang Shuren University, Hangzhou 310015, China; 3 Psychological Research and Counseling Center, Southwest Jiaotong University, Chengdu 611756, China

**Keywords:** growth mindset, family support, general self-efficacy, longitudinal study, Chinese children

## Abstract

Extensive research has highlighted the benefits of a growth mindset in fostering positive outcomes for children. However, the antecedents of children’s growth mindset of intelligence remain less known. This longitudinal study aimed to examine whether, and through which mechanism, perceived family support predicts children’s growth mindset over time. A total of 1874 Chinese children (*M*_age_ = 10.76 years, *SD* = 0.93) participated in this study, and they were evaluated twice, six months apart. In each wave, the children completed questionnaires, including a perceived family support scale, a general self-efficacy scale, and a growth mindset of intelligence scale. The results indicated that perceived family support can positively predict an increase in children’s growth mindset. Moreover, the longitudinal link between perceived family support and a growth mindset was mediated by children’s self-efficacy. Specifically, children’s perceived family support can positively predict an increase in self-efficacy, which in turn promotes a growth mindset. The findings highlight the significance of a supportive family environment for children’s intelligence mindset and that general self-efficacy can be a targeted intervention for improving their growth mindset.

## 1. Introduction

The growth mindset of intelligence (also known as growth mindset) is defined as the belief that intelligence is changeable and can be developed through effort and learning (Dweck et al., 1995). The significance of a growth mindset for individuals’ developmental outcomes, especially in the educational domain (Rege et al., 2021; Yeager & Walton, 2011), has been highlighted for eras, and research on this topic has yielded a variety of valuable findings (Dweck & Yeager, 2019). Although considerable progress has been made, some new issues are still worth exploring. In fact, more emphasis has been placed on how the growth mindset functions, but little attention has been given to how the growth mindset is formed. In other words, how do children develop a growth mindset rather than a fixed mindset?

In recent years, research has sought to identify the factors that can help develop a growth mindset (Haimovitz & Dweck, 2017). Children are able to form growth mindsets through a series of specific socialization practices (Dweck, 2017), such as praise from significant others and parental views and feedback about failure and success. Together, these specific socialization practices could contribute to a supportive context in which a growth mindset “seed” can be well developed (Walton & Yeager, 2020; Yeager & Dweck, 2020). As such, it is suggested that family support may be a main source for the development of a growth mindset.

However, among those related studies, two main limitations should be properly addressed. First, only a few cross-sectional studies among university students have provided direct support for the above suggested relationship; evidence from child samples and longitudinal data is scarce. Second, little is known about why children in a supportive family environment develop a growth mindset. To date, self-efficacy is well-developed in a context characterized by caring and warmth, which is supposedly a mediating mechanism through which perceived family support is related to children’s self-efficacy, and empirical evidence should be explicitly presented.

### 1.1. Perceived Family Support and the Growth Mindset of Intelligence

Previous research has revealed the positive effects of a growth mindset on children’s development across academic, cognitive, behavioral and emotional fields (Dweck & Yeager, 2019; King & Wang, 2025; Park et al., 2020; Yeager & Dweck, 2012). As such, cultivating children’s growth mindset can benefit them in the long-term. It is suggested that emphasis should be placed on the family environment (e.g., parenting practices), which may contribute to a growth mindset (Haimovitz & Dweck, 2017). Perceived family support may be such a factor that play a critical role in fostering a growth mindset of intelligence. Specifically, the perception of family support plays a vital role in overall social support; it involves the emotional, informational, or instrumental support that an individual subjectively feels from their relationships with family members (Cohen & Wills, 1985; Robertson, 1988).

Previous research has suggested that a lack of family support may be correlated with maladjustment outcomes (Koopmans et al., 2023). In contrast, higher levels of family support may indicate that abundant psychological resources are offered to help children cope with difficulties and challenges (Bradley, 2024; Parcel & Bixby, 2016). As such, a child in such a family context is more likely to be encouraged to embrace difficulties, seek process feedback, and persist through setbacks, all of which are hallmarks of a growth mindset profile (Altikulaç et al., 2024). Indeed, previous evidence has shown that children who grow up in an unsupportive family context are more likely to experience parental unconstructive educational involvement (Moorman & Pomerantz, 2010) and a lack of learning ability feedback (Barger et al., 2022), both of which are factors that are negatively correlated with a growth mindset of intelligence (Haimovitz & Dweck, 2017). In contrast, a supportive family context may be positively related to a growth mindset. For example, research using cross-sectional data has revealed that higher levels of perceived parental support correlate with increased growth mindset among university students (Lan et al., 2019; X. Wang & Wang, 2024).

Taken together, although previous research has revealed correlations between perceived family support and a growth mindset, two main limitations still need to be answered. First, although the abovementioned studies provide indirect support for the positive correlations between family support and a growth mindset among university students, few studies have examined the relationships among targeted children. Second, previous cross-sectional evidence can reveal only the correlation between perceived family support and growth mindset but cannot reveal the predictive direction of the two. As such, this study explicitly examines the relationship between children’s perceived family support and growth mindset by using longitudinal data.

### 1.2. Role of Self-Efficacy in Linking Perceived Family Support to Growth Mindset

Self-efficacy is the belief in one’s ability to perform particular actions (Bandura, 1999). It reflects confidence in one’s ability to exert control over his or her motivation, behavior, and environment (Bandura, 1978; Bandura et al., 1996). Research has demonstrated that self-efficacy positively impacts numerous developmental outcomes (Luszczynska et al., 2005), such as coping ability (Poluektova et al., 2023), happiness (F. Zhang & Yang, 2025), academic performance (Honicke & Broadbent, 2016), and mental health (Gallagher et al., 2020). More importantly, self-efficacy may serve as a critical mechanism through which perceived family support is correlated with a growth mindset.

On the one hand, a positive correlation may exist between children’s perceived family support and their self-efficacy levels. The family has long been regarded as the primary socialized context for children’s self-efficacy or competence beliefs, in which the main family members (i.e., parents) play a crucial role (Grolnick & Ryan, 1989; Grolnick & Slowiaczek, 1994). Within the social cognitive theory framework of Bandura (1997), supportive family settings may nurture both vicarious learning and social persuasion, which are key to developing self-efficacy. Specifically, children may experience more vicarious experiences through interactions with their parental role models and gain more encouragement from family members simultaneously (Usher & Pajares, 2008), both of which can help children internalize the belief that challenges are surmountable, thereby enhancing self-efficacy. In line with this evidence, empirical studies have revealed that perceived family support is positively correlated with self-efficacy. The majority of earlier studies have focused on the positive association between perceived familial support and self-efficacy among adolescents (Asici et al., 2024; Kleppang et al., 2023). Despite the evidence focused on adolescents, several studies focused on children have reported similar results. For example, a cross-sectional study reported that children’s perceived family support positively correlated with their self-efficacy in the academic domain (Liu et al., 2019). Moreover, when children reported better interactions and communication with their parents, their self-efficacy across different aspects was greater (Lv et al., 2018). In addition, a short-term longitudinal study revealed that children’s perceived parental warmth could positively predict their competence beliefs (Dinkelmann & Buff, 2016). Thus, it is reasonable that perceived family support can be positively correlated with children’s self-efficacy.

Alternatively, it is suggested that those with higher self-efficacy tend to demonstrate stronger growth mindsets about intelligence, and previous related research has provided support for this suggestion. Specifically, a recent study using cross-sectional data indicated that university students who reported higher levels of self-efficacy in their academics also reported higher levels of a growth mindset of intelligence (Zander et al., 2018). Similarly, individuals who possess stronger self-efficacy are more likely to endorse the view that intelligence is flexible and improvable through dedicated effort (Komarraju & Nadler, 2013). Despite the above two pieces of evidence, both of which focus primarily on university students and indicate a positive correlation between self-efficacy and intelligence mindset, there is limited evidence regarding this association among children. As such, this study aimed to address this issue.

To date, less evidence has explicitly examined this mediating role of self-efficacy. Relatedly, a recent study adopted self-efficacy as an indicator of a composite positive personality concept and revealed its mediating role in the relationship between perceived social support and intelligence growth mindset (L. Chen et al., 2024). Although this research did not examine the individual role of self-efficacy, considering the strong correlations between self-efficacy and the other two indicators that contribute to the composite positive personality concept, it is possible that self-efficacy could be a significant mediator. Therefore, when these two lines of evidence are combined, children’s self-efficacy may serve as a mediating factor linking perceived family support to the development of a growth mindset. In other words, adequate support from important family members may create a caring and warm environment for children to explore, which may be a crucial factor in facilitating children’s sense of competence and ability to cope with new challenges; in turn, reinforced self-efficacy is helpful for cultivating the conviction that intelligence can be enhanced through dedicated effort. However, although self-efficacy may act as such a mediator, less evidence has explicitly examined this mediating mechanism among children by using longitudinal data.

### 1.3. Current Study

The present research sought to examine whether perceived family support predicts increased growth mindset over time and the mediating mechanism of self-efficacy underlying this link among children. In particular, this study investigated these associations in the Chinese context.

Previous research on the antecedents of a growth mindset has focused predominantly on Western contexts (Haimovitz & Dweck, 2017; Hecht et al., 2021), and the findings may not be directly generalized to other social backgrounds; findings from Chinese society therefore help understand how to cultivate children’s growth mindset in non-Western countries. More importantly, China has a social background in which efforts, diligence and hard work are heavily emphasized through traditional cultural values (Li, 2003). All of these factors are closely correlated with a growth mindset (Dweck, 1999), making Chinese society an ideal environment in which to explore the factors that socialize and shape the growth mindset. Meanwhile, Chinese families have always been known for investing in many practices related to the learning and education of younger generations (Ng & Wei, 2020), which may help establish a nurturing family atmosphere conducive to cultivating a growth mindset. However, a recent study has indicated that Chinese children have a relatively low growth mindset compared with their American peers (Sun et al., 2021), which raises questions about whether this massive support in Chinese families can be effectively translated into a growth mindset. In this way, empirical evidence is desperately needed to explore this issue, and justifying the critical components that enable the establishment of a growth mindset in Chinese children may be helpful for parenting practices and education.

Therefore, by using two-time-point data, this research was designed to examine the impact of perceived familial support on growth mindset among children in China. This approach can help clarify the direction of the effect of perceived familial support on a growth mindset or whether there are bidirectional relationships. Moreover, this study aimed to reveal the mediating mechanism through which perceived family support longitudinally predicts Chinese children’s growth mindset. Specifically, although the significance of self-efficacy and related evidence indicate its potential mediating role (L. Chen et al., 2024), little evidence has revealed whether self-efficacy could mediate the longitudinal link between perceived familial support and children’s growth mindset. Taken together, the current longitudinal study aimed to investigate whether perceived family support is positively correlated with a growth mindset and whether the indirect effects of perceived family support on a growth mindset through self-efficacy are significant among Chinese children.

## 2. Materials and Methods

### 2.1. Participants

The study sample included students in third to fifth grades from three public elementary schools located in southeastern China via convenience sampling. Specifically, initial data collection (Time 1; T1) was conducted during the spring semester with 2084 participants aged 9–12 years (*M*_age_ = 10.74 years, *SD* = 0.93, 54.20% boys). About six months later, a follow-up assessment (Time 2; T2) was administered in the following fall semester with all original participants invited for retesting.

Approximately 11.37% of the children (*n* = 237) dropped out of the study at the two time points because of incomplete or partially completed questionnaires. Among the attrition data, 63.30% were boys (*n* = 150), 34.20% were in the third grade (*n* = 81), 32.90% were in the fourth grade (*n* = 78), and 32.90% were in the fifth grade (*n* = 78). To determine whether the measured variables at wave 1 differed significantly between the attritional sample and the non-attritional sample, independent samples t-test analyses were carried out. The results shown that the attrition sample had lower scores for perceived family support than did the non-attritional sample (*M*_attrition_ = 4.83, *SD* = 1.53; *M*_non-attrition_ = 5.07, *SD* = 1.42; *t* (2062) = −2.43, *p* = 0.015). There were no differences in self-efficacy (*M*_attrition_ = 2.59, *SD* = 0.59; *M*_non-attrition_ = 2.64, *SD* = 0.55; *t* (2041) = −1.52, *p* = 0.131) or growth mindset (*M*_attrition_ = 3.98, *SD* = 1.18; *M*_non-attrition_ = 4.04, *SD* = 1.16; *t* (2077) = −0.81, *p* = 0.418) between these two samples.

Finally, the valid data included 1847 children at both waves, which consisted of 980 boys and 867 girls with a mean age of 10.76 years at T1 (*SD* = 0.93). Moreover, among the valid participants, 525 children were in fourth grade, 678 children were in fifth grade, and the remaining 644 children were in sixth grade. The final valid sample (*n* = 1847) at both waves was therefore used in the subsequent data analyses.

### 2.2. Procedure

Prior to participation, written informed consent was obtained from both the participating children and their parents. Moreover, the study procedures received official approval from all participating school administrations. This was a longitudinal study, and the data were obtained directly from children’s self-reports through a paper-and-pencil questionnaire on a weekend in a quiet classroom setting. During the spring semester baseline assessment (Wave 1), the children were invited to participate in this study and completed questionnaires on their demographic information, perceived family support, self-efficacy and growth mindset. The follow-up assessment (Wave 2) in the subsequent fall semester involved readministering the same battery of measures. In addition, all items for the measurement scales can be found in Appendix A. As a token of appreciation, all participants received small gifts after completing each wave of data collection.

### 2.3. Measures

#### 2.3.1. Perceived Family Support

Children’s perceived family support was measured by the subscale of the Multidimensional Scale of Perceived Social Scale (MSPSS) (Zimet et al., 1988). The four items measuring perceived family support were used in the current study (i.e., “My family really tries to help me”, “I get the emotional help and support I need from my family”, “I can talk about my problems with my family”, and “My family is willing to help me make decisions”). This scale has been widely used in previous research and has demonstrated high reliability in Chinese samples (Lee & Mendoza, 2025). In both waves, the children provided ratings on a 7-point Likert scale (1 = strongly disagree to 7 = strongly agree). Composite scores were derived by averaging item responses, where higher scores reflected greater perceived family support. The internal consistency estimates were α = 0.77 at Wave 1 and α = 0.84 at Wave 2.

#### 2.3.2. Self-Efficacy

Children’s self-efficacy was measured by a widely used tool, i.e., the general self-efficacy scale (Schwarzer et al., 1999). This scale involves 10 items and employs a 4-point response format (1 = not at all true to 4 = completely true), including items such as “When I am confronted with a problem, I can usually find several solutions”. This scale is a widely validated tool across cultures (Luszczynska et al., 2005) and has demonstrated high reliability in Chinese samples (F. Zhang & Yang, 2025). In both waves, the children completed these ratings. The average scores across items served as indicators of self-efficacy levels, with higher scores representing higher levels of children’s self-efficacy. The measure demonstrated adequate internal consistency (α = 0.80 at Wave 1; α = 0.88 at Wave 2).

#### 2.3.3. Growth Mindset

Growth mindset was measured by the short-version implicit theory of intelligence scale (Dweck et al., 1995). This tool has been validated (Rammstedt et al., 2024), and the items were previously used with Chinese children (T. Zhang et al., 2022). In both waves, the children completed a total of three items (e.g., “Your intelligence is something about you that you can’t change very much”) on a 6-point Likert scale (1 = strongly agree to 6 = strongly disagree). The mean scores of all the items were calculated, with higher scores indicating a stronger growth mindset. The internal consistency estimates were α = 0.78 at Wave 1 and α = 0.84 at Wave 2.

### 2.4. Data Analyses

Statistical analyses were performed using SPSS 25.0 (IBM Corporation, NY) and Mplus 8.3 software packages. Preliminary analyses included Harman’s single-factor test, which revealed that the first factor explained 28.74% and 37.66% of the variance at Time 1 and Time 2, respectively, suggesting that common method bias was not a substantial concern in either assessment waves.

Descriptive statistics, including the means, standard deviations and correlations of the study variables, were calculated via SPSS 25.0. A total of three models were subsequently established and tested via Mplus 8.3. Throughout the data analyses, the missing data were addressed via the maximum likelihood estimation method, and 5000 bootstrapping replications with bias-adjusted 95% confidence intervals. Following established conventions, good model fit required CFI (i.e., comparative fit index) and TLI (i.e., Tucker–Lewis index) values above 0.90 and RMSEA (i.e., root mean square error of approximation) below 0.08.

First, a cross-lagged panel model was established to test whether perceived family support was reciprocally correlated with growth mindset. Second, a mediation model with the T2 growth mindset or family support as the independent variable was tested to determine the mediating role of self-efficacy. When the *p* value was significant (*p* < 0.05) and the confidence interval did not include a zero, the mediation models were considered effective. Specifically, two models were tested: One model was established to determine whether family support positively predicted growth mindset through self-efficacy overtime, and the other model was established to examine whether a growth mindset positively predicted perceived family support through self-efficacy over time. In addition, the hypothesized model was tested with the gender and T1 age controlled; gender served as a dummy-coded variable (boys = 0, girls = 1), with age standardized to z-scores. All additional continuous measures were standardized by converting to z-scores for subsequent analyses.

## 3. Results

### 3.1. Descriptive Statistics and Correlations Among the Main Variables

Descriptive statistics and bivariate correlations for all variables across the two waves are summarized in Table 1. At T1, perceived family support was significantly and positively related to self-efficacy (*r* = 0.38, *p* < 0.001) and a growth mindset (*r* = 0.28, *p* < 0.001); self-efficacy was positively related to a growth mindset (*r* = 0.41, *p* < 0.001). At T2, perceived family support was positively related to self-efficacy (*r* = 0.39, *p* < 0.001) and a growth mindset (*r* = 0.31, *p* < 0.001); self-efficacy was positively related to a growth mindset (*r* = 0.53, *p* < 0.001).

Moreover, the results revealed that T1 perceived family support was positively correlated with T2 perceived family support (*r* = 0.42, *p* < 0.001); T1 self-efficacy was positively related to T2 self-efficacy (*r* = 0.46, *p* < 0.001); and T1 growth mindset was positively related to T2 growth mindset (*r* = 0.40, *p* < 0.001).

In addition, perceived family support at T1 was positively related to T2 self-efficacy (*r* = 0.28, *p* < 0.001) and T2 growth mindset (*r* = 0.23, *p* < 0.001). Self-efficacy at T1 was positively correlated with having a growth mindset at T2 (*r* = 0.32, *p* < 0.001).

### 3.2. Cross-Lagged Model of the Link Between Perceived Family Support and Growth Mindset

**Figure 1:**
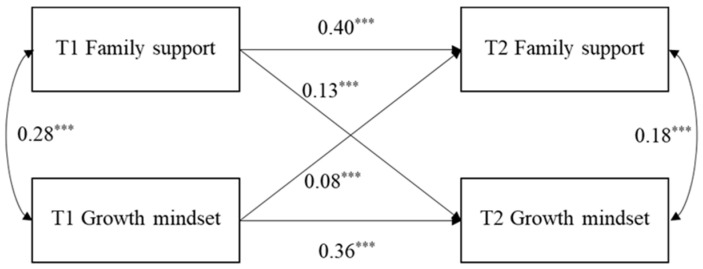
Cross-lagged model of perceived family support and growth mindset. (Note: gender and T1 age were controlled for in the model and are not shown for simplicity). *** *p* < 0.001.

The cross-lagged model of the relationships between perceived family support and growth mindset is shown in Figure 1.

The model fit indices were adequate as follows: χ^2^_(4)_ = 16.67, *p* = 0.002; CFI = 0.984, TLI = 0.965, RMSEA = 0.04 [0.02, 0.06], and SRMR = 0.02. Specifically, the results showed that perceived family support at T1 positively and significantly predicted T2 growth mindset (β = 0.13, SE = 0.02, *p* < 0.001, 95% CI = [0.09, 0.17]). In addition, having a growth mindset at T1 significantly and positively predicted T2 perceived family support (β = 0.08, SE = 0.02, *p* < 0.001, 95% CI = [0.04, 0.13]).

### 3.3. Mediation Model for T2 Growth Mindset

A mediation analysis was conducted to determine if self-efficacy mediated the longitudinal relationship between perceived family support at T1 and growth mindset at T2.

**Figure 2:**
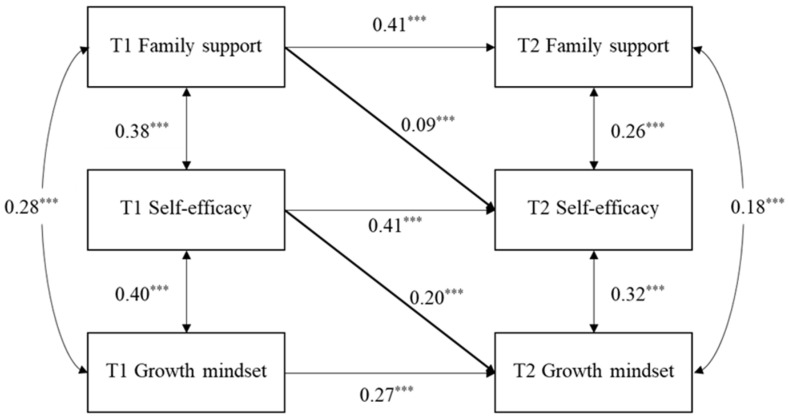
T1 perceived family support predicts T2 growth mindset through self-efficacy. (Note: Children’s gender and T1 age are controlled for but not shown for simplicity). *** *p* < 0.001.

The model fit indices were adequate as follows: χ^2^_(14)_ = 74.68, *p* < 0.001; CFI = 0.967, TLI = 0.958, RMSEA = 0.05 [0.04, 0.06], and SRMR = 0.03. Specifically, as shown in Figure 2, the results showed that perceived family support positively predicted T2 self-efficacy (β = 0.09, SE = 0.02, *p* < 0.001, 95% CI = [0.05, 0.13]), which in turn positively predicted T2 growth mindset (β = 0.20, SE = 0.02, *p* < 0.001, 95% CI = [0.16, 0.25]). Thus, self-efficacy significantly mediated the positive link between T1 perceived family support and T2 growth mindset.

### 3.4. Mediation Model for T2 Perceived Family Support

Additionally, a mediation analysis was also conducted with T2 perceived family support as the outcome, examining whether self-efficacy mediated the effect of T1 growth mindset on later perceived family support.

**Figure 3:**
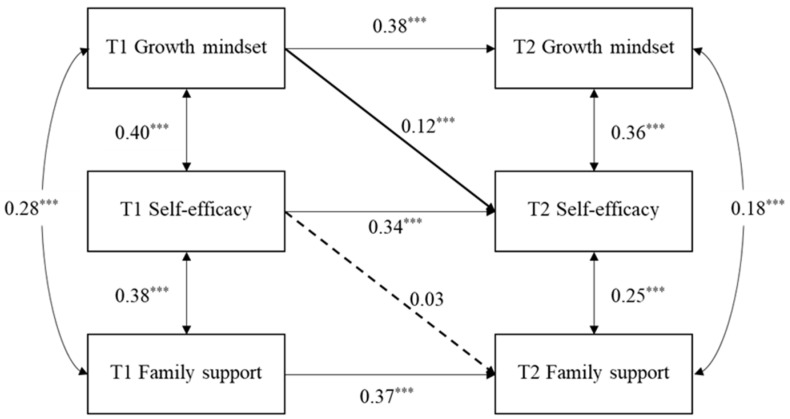
T1 growth mindset predicts T2 perceived family support through self-efficacy. (Note: Children’s gender and T1 age are controlled for but not shown for simplicity; the dashed line indicates non-significance at *p* value greater than 0.05). *** *p* < 0.001.

The model fit indices are as follows: χ^2^_(14)_ = 140.36, *p* < 0.001; CFI = 0.931, TLI = 0.912, RMSEA = 0.06 [0.05, 0.08], and SRMR = 0.05. Referring to Figure 3, the results revealed that the growth mindset at T1 positively predicted self-efficacy at T2 (β = 0.12, SE = 0.02, *p* < 0.001, 95% CI = [0.08, 0.17]).

However, T2 self-efficacy did not significantly predict T2 perceived family support (β = 0.03, SE = 0.02, *p* = 0.132, 95% CI = [−0.02, 0.08]). As such, self-efficacy cannot mediate the positive relationship between a T1 growth mindset and T2 perceived family support.

## 4. Discussion

While the effects of a growth mindset are well established in the educational psychology literature, the developmental antecedents remain insufficiently delineated, particularly within Chinese populations. This study included Chinese school-aged children as the target participants and explicitly investigated the longitudinal relationship between perceived family support and growth mindset and the mediating role of self-efficacy in this relationship. As a result, this study revealed that perceived family support positively predicts increased growth mindset among Chinese children. Additionally, self-efficacy could mediate the longitudinal link between perceived family support and children’s growth mindset, suggesting that the positive effect of family support on intelligence mindset operates partially through enhanced self-efficacy among Chinese children.

These findings are in line with social cognitive theory (Bandura, 1997) and highlight the function of self-efficacy in linking the longitudinal relationship between perceived family support and a growth mindset. Family support was conducive to children’s self-efficacy, which in turn facilitated their belief in intelligence as a malleable trait. More importantly, these findings have extended mindset theory (Dweck, 1999; Dweck & Leggett, 1988) and have explicitly revealed the factors that contribute to the development of a growth mindset in a nonwestern social context. Instead of focusing on developmental outcomes in relation to the growth mindset (Blackwell et al., 2007; Dweck et al., 1995; Dweck & Yeager, 2019), this study, in accordance with recent research focused on the origin of the growth mindset (Haimovitz & Dweck, 2017), helps understand how a growth mindset among Chinese children can be facilitated.

This study revealed the positive effects of perceived family support on children’s growth mindset over time. These findings support the view that the socialization context is important for shaping a growth mindset (Kim, 2023). This evidence was in line with previous cross-sectional studies indicating positive correlations between perceived family support and growth mindset (Lan et al., 2019; X. Wang & Wang, 2024). Moreover, the findings of this study further indicate that perceived family support positively predicts a growth mindset, whereas a growth mindset cannot predict perceived family support. Consistent with the ecological model of human development (Bronfenbrenner, 1979) and the bioecological model of human development (Bronfenbrenner & Morris, 2006), children’s personal characteristics (e.g., intelligence mindset) may be constantly shaped by their proximal process (e.g., parenting practices). As this study indicated, children who perceive higher levels of family support show an increased propensity to view intelligence as improvable. In other words, children’s growth mindset is shaped and facilitated by family support. As such, in addition to those specific parenting practices and beliefs (Dweck, 2017; Haimovitz & Dweck, 2017), the supportive family atmosphere that children perceived could also cultivate their intelligence mindset. This finding is also consistent with recent studies, which have indicated that when sufficient needs and support are adequate, a growth mindset is more likely to develop (Walton & Yeager, 2020; Yeager et al., 2022). Indeed, when children encounter challenging tasks or adverse circumstances, emotional support and informal guidance from family members can potentially help them manage and navigate these stressors (Heerde & Hemphill, 2018), which directly contributes to cognitive development (Parcel & Bixby, 2016).

This longitudinal study identifies self-efficacy as a key mediating factor through which perceived family support influences the cultivation of a growth mindset in Chinese children. As such, this finding highlights the underlying mechanism by which perceived family support is linked with children’s growth mindset. This finding extends previous evidence from cross-sectional studies that focused on adolescents (Asici et al., 2024; Kleppang et al., 2023) and revealed positive effects of perceived family support on children’s self-efficacy. Moreover, this finding has added direct evidence to the link between self-efficacy and children’s intelligence mindset. In fact, few studies exploring the correlations between self-efficacy and growth mindset have focused on university students (Komarraju & Nadler, 2013; Zander et al., 2018); thus, this finding helps provide new evidence for understanding this relationship among Chinese children. Specifically, when children perceive higher levels of support provided by family members, they develop greater self-efficacy in coping with difficulties and challenging tasks. This enhanced self-efficacy then contributes to an increase in their beliefs about the changeability of intelligence.

For young children, a constructive and connected relationship with significant family members is vital for academic and psychological success (Brumariu & Kerns, 2010). Adequate information and warmth from family members are related to less maladaptive coping strategies (Gervais & Jose, 2024), which might help children deal with a variety of situations more successfully. According to social cognitive theory (Bandura, 1997), these successful experiences can directly and gradually cultivate children’s confidence in their ability to handle challenging assignments. As indicated by previous research (Begen & Turner-Cobb, 2015), perceived family support might meet children’s need for belonging and therefore enhance their self-esteem, a concept related to self-efficacy (G. Chen et al., 2004). Moreover, a meta-analysis study has indicated a moderate-to-strong correlation between self-efficacy and mastery approach goals (Huang, 2016). Thus, highly self-efficacious children may seek approach goals that emphasize their learning processes. As suggested by previous research (Dweck, 1999), mastery or learning goals can constantly and positively shape children’s growth mindset. In this way, through the pursuit of these learning goals, children acquire new skills and competencies; this process enables them to understand that their capacities are malleable and subject to growth, thereby reinforcing their formation of a growth mindset. Therefore, children’s perceived family support enhances their growth mindset both directly and indirectly through improved self-efficacy.

## 5. Limitations and Directions for Future Research

This research has certain limitations that warrant consideration. First, the current study did not explicitly address family autonomy support. This concept, a more specific type of family support practice, is also known to contribute to an individual’s motivation (Vasquez et al., 2016). The results of the current study may have limited generalizability to research focused on family autonomy support. Future research could also explicitly aim at family autonomy support to provide more understanding of its role in children’s development of self-efficacy and intelligence growth mindset.

Second, related to the first point, family support is only one of the social support sources, and the implications of the other two sources (i.e., teachers and peers) of social support were suggested simultaneously in previous research (X. Wang & Wang, 2024); thus, the findings of this study could not fully reflect the effects of children’s social support. Indeed, teachers’ support for a growth mindset also plays a vital role in facilitating the development of this mindset in children (Vestad & Bru, 2024). Future research could focus on all three sources of social support and reveal either the overall effects or the individual effects of family and school (e.g., teachers) support.

Third, this study did not collect or control for any information about children’s primary family members (e.g., parents), which may have an impact on the main results. For example, previous research has revealed that family socioeconomic status may be correlated with parents’ practices in children’s learning (F. Zhang et al., 2020). In this way, future research could pay attention to and control for several factors related to family background, thus providing a fuller understanding of the effects of perceived family support on children’s motivation development.

Fourth, this study focused on children’s perceived family support, and the data were collected through children’s reports. Meanwhile, the family support measured in this study refers broadly to support from all family members and not solely from parents. Taken together, an interesting avenue for future research would be to collect reports on family support from primary figures (e.g., parents). This could help examine the extent to which parent-provided support contributes to children’s growth mindset or to investigate the effects of parent–child support discrepancies on child development.

Finally, although this study revealed the positive effects of perceived family support, excessive family support without appropriate control may hinder the development of motivation (Huver et al., 2010), especially in Chinese culture (Ng & Wei, 2020). Thus, it is necessary to prevent excessive parental support from transforming into indulgent parenting, which may inadvertently cause impairment.

## 6. Implications

The implications of the current study could be well elaborated. First and foremost, these findings provide new empirical evidence for mindset theory (Dweck et al., 1995) by revealing critical components enabling the establishment of growth mindset. Specifically, this study highlights the benefits of a supportive family environment in facilitating a growth mindset in Chinese culture. Given that previous suggestions on the antecedents of a growth mindset were predominantly based on the Western population (Dweck, 2017; Haimovitz & Dweck, 2017), the findings of the current study can be used to understand how Chinese children’s growth mindset has developed.

Moreover, previous research has suggested that Chinese children possess lower levels of a growth mindset than their American peers (Sun et al., 2021). Some studies have argued that cultural beliefs play a more important role in Chinese children’s developmental outcomes than their growth mindset does (Li, 2003, 2005; van Egmond et al., 2013), a conclusion that could weaken the perceived benefits of a growth mindset in Chinese culture. Despite this argument, the positive function of a growth mindset still exists in China (Q. Wang & Ng, 2012; Y. Wang & Sun, 2025). Therefore, cultivating the growth mindset of Chinese children may still be beneficial. The current study could help provide a practical basis for cultivating a growth mindset among Chinese children. In other words, future interventions aimed at improving Chinese children’s growth mindset could involve establishing a supportive family environment or increasing their self-efficacy, both of which are beneficial for Chinese children’s views on the malleability of intelligence.

## 7. Conclusions

This study revealed that perceived family support can positively predict an increase in Chinese children’s growth mindset over time. Moreover, self-efficacy mediated this short-term longitudinal relationship between perceived family support and growth mindset. The positive effects of perceived family support on growth mindset can be partly explained by self-efficacy among Chinese children.

## Figures and Tables

**Table 1:** Descriptive statistics and correlations for all variables.

Variables	*M*	*SD*	1	2	3	4	5	6	7
1. Perceived family support (T1)	5.05	1.44	—						
2. Self-efficacy (T1)	2.64	0.56	0.38 ***	—					
3. Growth mindset (T1)	4.04	1.16	0.28 ***	0.41 ***	—				
4. Perceived family support (T2)	5.12	1.49	0.42 ***	0.21 ***	0.20 ***	—			
5. Self-efficacy (T2)	2.68	0.61	0.28 ***	0.46 ***	0.29 ***	0.39 ***	—		
6. Growth mindset (T2)	3.87	1.21	0.23 ***	0.32 ***	0.40 ***	0.31 ***	0.53 ***	—	
7. Gender	0.46	0.50	0.03	−0.01	0.01	0.03	−0.01	−0.02	—
8. Age (T1)	10.76	0.93	−0.01	−0.004	−0.08 ***	−0.01	−0.06 **	−0.08 ***	−0.04

Note. *** *p* < 0.001, and ** *p* < 0.01; gender is a dummy variable (boys = 0, girls = 1).

## Data Availability

The data that support the findings of this study are available on request from the authors.

## Appendix A. Measurement Items

(Note: The Chinese version of the items was administered. The original English versions are provided below.)

**The four items that measure Perceived Family Support for children:**

1. My family really tries to help me.
2. I get the emotional help and support I need from my family.
3. I can talk about my problems with my family.
4. My family is willing to help me make decisions.

**The ten items measure Self-efficacy for children:**

1. I can always manage to solve difficult problems if I try hard enough.
2. if someone opposes me, I can find means and ways to get what I want.
3. it is easy for me to stick to my aims and accomplish my goals.
4. I am confident that I could deal efficiently with unexpected events.
5. Thanks to my resourcefulness, I know how to handle unforeseen situations.
6. I can solve most problems if I invest the necessary effort.
7. I can remain calm when facing difficulties because I can rely on my coping abilities.
8. When I am confronted with a problem, I can usually find several solutions.
9. If I am in trouble, I can usually think of something to do.
10. No matter what comes my way, I am usually able to handle it.

**The three items that measure Growth Mindset for children:**

1. You have a certain amount of intelligence, and you can’t really do much to change it.
2. Your intelligence is something about you that you can’t change very much.
3. You can learn new things, but you can’t really change your basic intelligence.

## References

[B1-behavsci-15-01182] Altikulaç S., Janssen T. W. P., Yu J., Nieuwenhuis S., Van Atteveldt N. M. (2024). Mindset profiles of secondary school students: Associations with academic achievement, motivation and school burnout symptoms. British Journal of Educational Psychology.

[B2-behavsci-15-01182] Asici E., Katmer A. N., Agca M. A. (2024). Linking perceived family and peer support to hope in Syrian refugee adolescents: The mediating role of academic self-efficacy. Child and Adolescent Social Work Journal.

[B3-behavsci-15-01182] Bandura A. (1978). Self-efficacy: Toward a unifying theory of behavioral change. Advances in Behaviour Research and Therapy.

[B4-behavsci-15-01182] Bandura A. (1997). Self-efficacy: The exercise of control.

[B5-behavsci-15-01182] Bandura A. (1999). Social cognitive theory: An agentic perspective. Asian Journal of Social Psychology.

[B6-behavsci-15-01182] Bandura A., Barbaranelli C., Caprara G. V., Pastorelli C. (1996). Multifaceted impact of self-efficacy beliefs on academic functioning. Child Development.

[B7-behavsci-15-01182] Barger M. M., Wu J., Xiong Y., Oh D. D., Cimpian A., Pomerantz E. M. (2022). Parents’ responses to children’s math performance in early elementary school: Links with parents’ math beliefs and children’s math adjustment. Child Development.

[B8-behavsci-15-01182] Begen F. M., Turner-Cobb J. M. (2015). Benefits of belonging: Experimental manipulation of social inclusion to enhance psychological and physiological health parameters. Psychol Health.

[B9-behavsci-15-01182] Blackwell L. S., Trzesniewski K. H., Dweck C. S. (2007). Implicit theories of intelligence predict achievement across an adolescent transition: A longitudinal study and an intervention. Child Development.

[B10-behavsci-15-01182] Bradley R. H., Troop-Gordon W., Neblett E. W. (2024). Home environment. Encyclopedia of adolescence.

[B11-behavsci-15-01182] Bronfenbrenner U. (1979). The ecology of human development: Experiments in nature and design.

[B12-behavsci-15-01182] Bronfenbrenner U., Morris P. A., Damon R. M. L. W. (2006). The bioecological model of human development. Handbook of child psychology: Theoretical models of human development.

[B13-behavsci-15-01182] Brumariu L. E., Kerns K. A. (2010). Parent–child attachment and internalizing symptoms in childhood and adolescence: A review of empirical findings and future directions. Development and Psychopathology.

[B14-behavsci-15-01182] Chen G., Gully S. M., Eden D. (2004). General self-efficacy and self-esteem: Toward theoretical and empirical distinction between correlated self-evaluations. Journal of Organizational Behavior.

[B15-behavsci-15-01182] Chen L., Zhang Y., Tang Y. (2024). The relationship between school climate and growth mindset of junior middle school students: A mediating model of perceived social support and positive personality. Current Psychology.

[B16-behavsci-15-01182] Cohen S., Wills T. A. (1985). Stress, social support, and the buffering hypothesis. Psychological Bulletin.

[B17-behavsci-15-01182] Dinkelmann I., Buff A. (2016). Children’s and parents’ perceptions of parental support and their effects on children’s achievement motivation and achievement in mathematics. A longitudinal predictive mediation model. Learning and Individual Differences.

[B18-behavsci-15-01182] Dweck C. S. (1999). Self-theories: Their role in motivation, personality, and development.

[B19-behavsci-15-01182] Dweck C. S. (2017). The journey to children’s mindsets—And beyond. Child Development Perspectives.

[B22-behavsci-15-01182] Dweck C. S., Chiu C.-Y., Hong Y.-Y. (1995). Implicit theories and their role in judgments and reactions: A word from two perspectives. Psychological Inquiry.

[B20-behavsci-15-01182] Dweck C. S., Leggett E. L. (1988). A social-cognitive approach to motivation and personality. Psychological Review.

[B21-behavsci-15-01182] Dweck C. S., Yeager D. S. (2019). Mindsets: A view from two eras. Perspectives on Psychological Science.

[B23-behavsci-15-01182] Gallagher M. W., Long L. J., Phillips C. A. (2020). Hope, optimism, self-efficacy, and posttraumatic stress disorder: A meta-analytic review of the protective effects of positive expectancies. Journal of Clinical Psychology.

[B24-behavsci-15-01182] Gervais C., Jose P. E. (2024). Relationships between family connectedness and stress-triggering problems among adolescents: Potential mediating role of coping strategies. Research on Child and Adolescent Psychopathology.

[B25-behavsci-15-01182] Grolnick W. S., Ryan R. M. (1989). Parent styles associated with children’s self-regulation and competence in school. Journal of Educational Psychology.

[B26-behavsci-15-01182] Grolnick W. S., Slowiaczek M. L. (1994). Parents’ involvement in children’s schooling: A multidimensional conceptualization and motivational model. Child Development.

[B27-behavsci-15-01182] Haimovitz K., Dweck C. S. (2017). The origins of children’s growth and fixed mindsets: New research and a new proposal. Child Development.

[B28-behavsci-15-01182] Hecht C. A., Yeager D. S., Dweck C. S., Murphy M. C. (2021). Beliefs, affordances, and adolescent development: Lessons from a decade of growth mindset interventions. Advances in Child Development and Behavior.

[B29-behavsci-15-01182] Heerde J. A., Hemphill S. A. (2018). Examination of associations between informal help-seeking behavior, social support, and adolescent psychosocial outcomes: A meta-analysis. Developmental Review.

[B30-behavsci-15-01182] Honicke T., Broadbent J. (2016). The influence of academic self-efficacy on academic performance: A systematic review. Educational Research Review.

[B31-behavsci-15-01182] Huang C. (2016). Achievement goals and self-efficacy: A meta-analysis. Educational Research Review.

[B32-behavsci-15-01182] Huver R. M. E., Otten R., de Vries H., Engels R. C. M. E. (2010). Personality and parenting style in parents of adolescents. Journal of Adolescence.

[B33-behavsci-15-01182] Kim M. H. (2023). A bioecological perspective on mindset. Contemporary Educational Psychology.

[B34-behavsci-15-01182] King R. B., Wang F. M. (2025). The rich get richer: Socioeconomic advantage amplifies the role of growth mindsets in learning. British Journal of Educational Psychology.

[B35-behavsci-15-01182] Kleppang A. L., Steigen A. M., Finbråten H. S. (2023). Explaining variance in self-efficacy among adolescents: The association between mastery experiences, social support, and self-efficacy. BMC Public Health.

[B36-behavsci-15-01182] Komarraju M., Nadler D. (2013). Self-efficacy and academic achievement: Why do implicit beliefs, goals, and effort regulation matter?. Learning and Individual Differences.

[B37-behavsci-15-01182] Koopmans Y., Nelemans S. A., Bosmans G., van den Noortgate W., Van Leeuwen K., Goossens L. (2023). Perceived parental support and psychological control, DNA methylation, and loneliness: Longitudinal associations across early adolescence. Journal of Youth and Adolescence.

[B38-behavsci-15-01182] Lan X., Ma C., Radin R. (2019). Parental autonomy support and psychological well-being in Tibetan and Han emerging adults: A serial multiple mediation model. Frontiers in Psychology.

[B39-behavsci-15-01182] Lee H. J., Mendoza N. B. (2025). Does parental support amplify growth mindset predictions for student achievement and persistence? Cross-cultural findings from 76 countries/regions. Social Psychology of Education.

[B40-behavsci-15-01182] Li J. (2003). U.S and Chinese cultural beliefs about learning. Journal of Educational Psychology.

[B41-behavsci-15-01182] Li J. (2005). Mind or virtue: Western and Chinese beliefs about learning. Current Directions in Psychological Science.

[B42-behavsci-15-01182] Liu Y., Sang B., Liu J., Gong S., Ding X. (2019). Parental support and homework emotions in Chinese children: Mediating roles of homework self-efficacy and emotion regulation strategies. Educational Psychology.

[B43-behavsci-15-01182] Luszczynska A., Gutiérrez-Doña B., Schwarzer R. (2005). General self-efficacy in various domains of human functioning: Evidence from five countries. International Journal of Psychology.

[B44-behavsci-15-01182] Lv B., Zhou H., Liu C., Guo X., Liu J., Jiang K., Liu Z., Luo L. (2018). The relationship between parental involvement and children’s self-efficacy profiles: A person-centered approach. Journal of Child and Family Studies.

[B45-behavsci-15-01182] Moorman E. A., Pomerantz E. M. (2010). Ability mindsets influence the quality of mothers’ involvement in children’s learning: An experimental investigation. Developmental Psychology.

[B46-behavsci-15-01182] Ng F. F.-Y., Wei J. (2020). Delving into the minds of Chinese parents: What beliefs motivate their learning-related practices?. Child Development Perspectives.

[B47-behavsci-15-01182] Parcel T. L., Bixby M. S. (2016). The ties that bind: Social capital, families, and children’s well-being. Child Development Perspectives.

[B48-behavsci-15-01182] Park D., Tsukayama E., Yu A., Duckworth A. L. (2020). The development of grit and growth mindset during adolescence. Journal of Experimental Child Psychology.

[B49-behavsci-15-01182] Poluektova O., Kappas A., Smith C. A. (2023). Using Bandura’s self-efficacy theory to explain individual differences in the appraisal of problem-focused coping potential. Emotion Review.

[B50-behavsci-15-01182] Rammstedt B., Grüning D. J., Lechner C. M. (2024). Measuring growth mindset: Validation of a three-item and a single-item scale in adolescents and adults. European Journal of Psychological Assessment.

[B51-behavsci-15-01182] Rege M., Hanselman P., Solli I. F., Dweck C. S., Ludvigsen S., Bettinger E., Crosnoe R., Muller C., Walton G., Duckworth A., Yeager D. S. (2021). How can we inspire nations of learners? An investigation of growth mindset and challenge-seeking in two countries. American Psychologist.

[B52-behavsci-15-01182] Robertson S. E. (1988). Social support: Implications for counselling. International Journal for the Advancement of Counselling.

[B53-behavsci-15-01182] Schwarzer R., Mueller J., Greenglass E. (1999). Assessment of perceived general self-efficacy on the internet: Data collection in cyberspace. Anxiety, Stress, & Coping.

[B54-behavsci-15-01182] Sun X., Nancekivell S., Gelman S. A., Shah P. (2021). Growth mindset and academic outcomes: A comparison of US and Chinese students. NPJ Science of Learning.

[B55-behavsci-15-01182] Usher E. L., Pajares F. (2008). Sources of self-efficacy in school: Critical review of the literature and future directions. Review of Educational Research.

[B56-behavsci-15-01182] van Egmond M. C., Kühnen U., Li J. (2013). Mind and virtue: The meaning of learning, a matter of culture?. Learning, Culture and Social Interaction.

[B57-behavsci-15-01182] Vasquez A. C., Patall E. A., Fong C. J., Corrigan A. S., Pine L. (2016). Parent autonomy support, academic achievement, and psychosocial functioning: A meta-analysis of research. Educational Psychology Review.

[B58-behavsci-15-01182] Vestad L., Bru E. (2024). Teachers’ support for growth mindset and its links with students’ growth mindset, academic engagement, and achievements in lower secondary school. Social Psychology of Education.

[B59-behavsci-15-01182] Walton G. M., Yeager D. S. (2020). Seed and soil: Psychological affordances in contexts help to explain where wise interventions succeed or fail. Current Directions in Psychological Science.

[B60-behavsci-15-01182] Wang Q., Ng F. F.-Y. (2012). Chinese students’ implicit theories of intelligence and school performance: Implications for their approach to schoolwork. Personality and Individual Differences.

[B61-behavsci-15-01182] Wang X., Wang Y. (2024). The impact of perceived social support on e-learning engagement among college students: Serial mediation of growth mindset and subjective well-being. European Journal of Psychology of Education.

[B62-behavsci-15-01182] Wang Y., Sun X. (2025). Growth mindset in Chinese culture: A meta-analysis. Social Psychology of Education.

[B66-behavsci-15-01182] Yeager D. S., Carroll J. M., Buontempo J., Cimpian A., Woody S., Crosnoe R., Muller C., Murray J., Mhatre P., Kersting N., Hulleman C., Kudym M., Murphy M., Duckworth A. L., Walton G. M., Dweck C. S. (2022). Teacher mindsets help explain where a growth-mindset intervention does and doesn’t work. Psychological Science.

[B63-behavsci-15-01182] Yeager D. S., Dweck C. S. (2012). Mindsets that promote resilience: When students believe that personal characteristics can be developed. Educational Psychologist.

[B64-behavsci-15-01182] Yeager D. S., Dweck C. S. (2020). What can be learned from growth mindset controversies?. American Psychologist.

[B65-behavsci-15-01182] Yeager D. S., Walton G. M. (2011). Social-psychological interventions in education: They’re not magic. Review of Educational Research.

[B67-behavsci-15-01182] Zander L., Brouwer J., Jansen E., Crayen C., Hannover B. (2018). Academic self-efficacy, growth mindsets, and university students’ integration in academic and social support networks. Learning and Individual Differences.

[B69-behavsci-15-01182] Zhang F., Jiang Y., Ming H., Ren Y., Wang L., Huang S. (2020). Family socio-economic status and children’s academic achievement: The different roles of parental academic involvement and subjective social mobility. British Journal of Educational Psychology.

[B68-behavsci-15-01182] Zhang F., Yang R. (2025). Parental expectations and adolescents’ happiness: The role of self-efficacy and connectedness. BMC Psychology.

[B70-behavsci-15-01182] Zhang T., Park D., Ungar L. H., Tsukayama E., Luo L., Duckworth A. L. (2022). The development of grit and growth mindset in Chinese children. Journal of Experimental Child Psychology.

[B71-behavsci-15-01182] Zimet G. D., Dahlem N. W., Zimet S. G., Farley G. K. (1988). The multidimensional scale of perceived social support. Journal of Personality Assessment.

